# Supplementary material for: Work-Function-Resolved Imaging of Relaxation Oscillations and Local Kinetic Heterogeneities in CO Oxidation over Platinum Surfaces
Source: Langmuir. 2026 Jun 26;42(27):20010–9. doi: 10.1021/acs.langmuir.6c02992 (PMC13374379; doi:10.1021/acs.langmuir.6c02992)
Supplement: Supplementary file 1 [file la6c02992_si_001.pdf]

Supporting Information for

Work-Function-Resolved Imaging of Relaxation Oscillations and Local Kinetic Heterogeneities in CO Oxidation over Platinum Surfaces

Karel Vařeka[1], Michal Potoček[1,2], Martin Kovařík[1], Adam Očkovič[2], Tomáš Šikola[1,2], Zhu-Jun Wang[3], Petr Bábor[1,2], Miroslav Kolíbal[1,2*]

[1]Brno University of Technology, Central European Institute of Technology, Purkyňova 123, 61200 Brno, Czech Republic

[2]Brno University of Technology, Faculty of Mechanical Engineering, Institute of Physical Engineering, Technická 2, 616 69 Brno, Czech Republic

[3]School of Physical Science and Technology, Shanghai Tech University, Shanghai, 201210, China

[*]kolibal.m@fme.vutbr.cz

Table of contents

Description of Supplementary Movies……………………………………………………….. 2

Supplementary Figure S1…………………………………………………………………...3

Supplementary Figure S2…………………………………………………………………...4

Movie M1: Video sequence of operando SEM showing spiral and planar reaction waves on single-crystal Pt(110) during catalytic CO oxidation. Spiral evolution observed at p(CO) = 4×10^{-4} Pa, p(O_2) = 1.5×10^{-3} Pa. T = 204 °C. The patterns consist of two distinct greyscale levels corresponding to different surface adsorbed species.

Movie M2: Video obtained during correlative SEM-KPFM on polycrystalline Pt at p(CO) = 3×10^{-3} Pa and p(O_2) = 1.6×10^{-2} Pa, T = 216 °C experimental conditions. The sequence shows adsorbate-induced wavefront evolving across the Pt grain close to the KPFM probe.

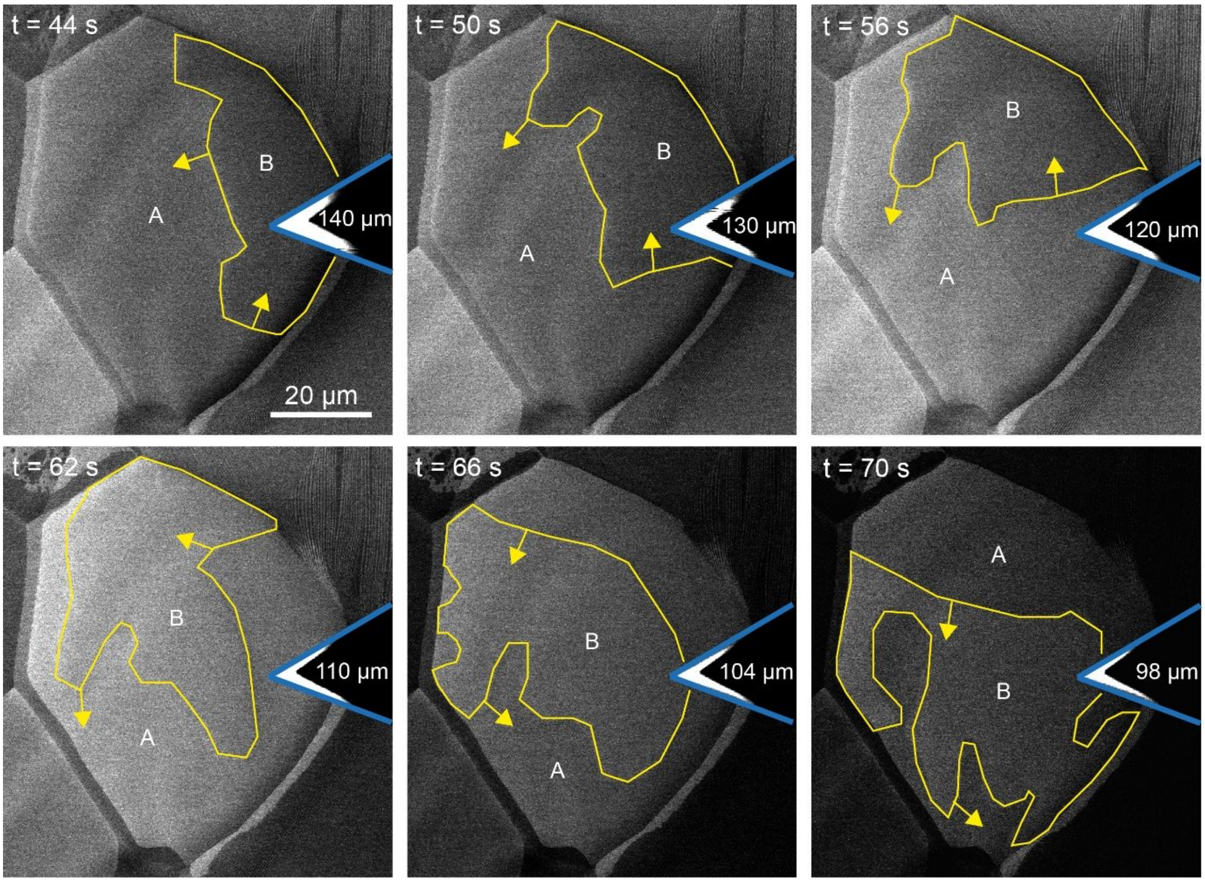

Movie M3: Adsorbate-induced contrast reversal during an AFM-tip approach to the polycrystalline Pt surface. Experimental conditions: p(CO) = 2.8×10^{-3} Pa, p(O_2) = 1.4×10^{-2} Pa, T ~ 210 °C (the temperature was continuously adjusted in order to compensate for sample cooling caused by the proximity of the tip). A dark-contrast reaction wave (marked with letter B) encircled in yellow changes the grey-level contrast with respect to the nearby bright-contrast reaction wave A during the tip approach. The contrast reversal occurs at around t = 62 s, when the tip-sample distance is approx. 110 µm.

Movie M4: Reaction-diffusion model video sequence showing the spiralling patterns of CO and oxygen waves on platinum. Each pixel corresponds to a potential adsorption site for O (red) or CO (green) on the catalyst (empty adsorption site is black).

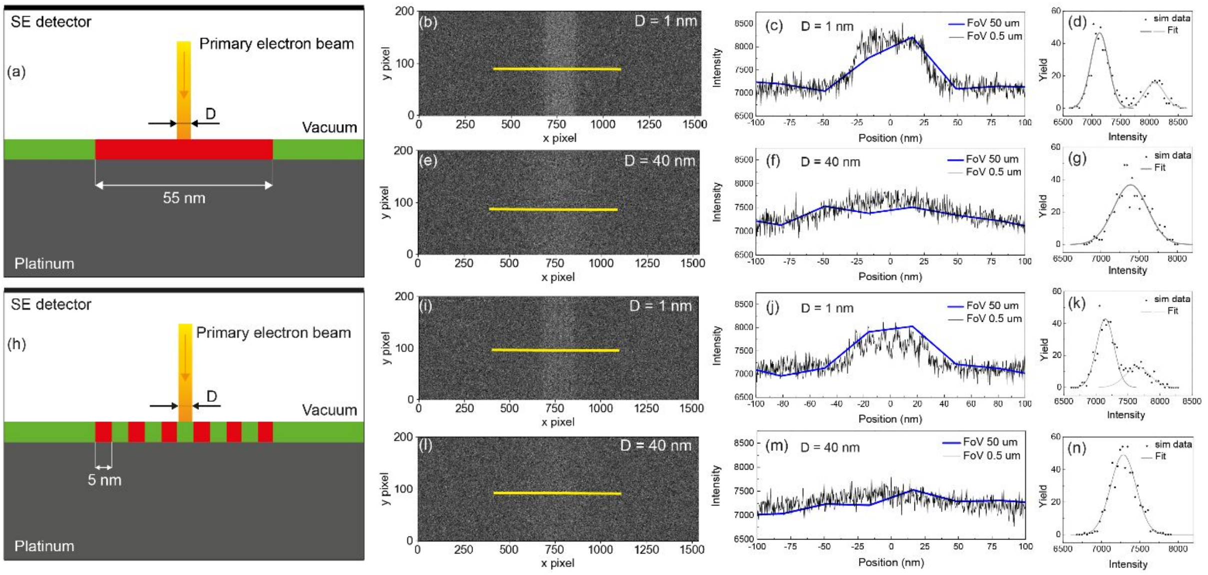

Fig. S1: Secondary electron images calculated by Monte-Carlo-based Nebula software. The simulator models an electron transport using first-principle physical scattering models, treating electrons as classical particles undergoing free movement between discrete elastic, inelastic, and boundary-crossing events. Elastic scattering is described by Mott cross sections (ELSEPA), while inelastic scattering is modeled using the full Penn dielectric function algorithm. Electron transmission and reflection at material interfaces are described by a quantum mechanical step-potential model. (a) Schematic representation of the simulation setup. A primary electron beam (energy 5 keV, beam fluence of 100 electrons/px) was directed at the Pt substrate featuring regions with different work functions to simulate CO and O coverage. The difference between the work-function values of each region (red and green areas in the schematic) was inferred from values reported in Refs. [30, 44] (main article). (b) Simulated SE image of 55 nm wide oxygen stripe on otherwise CO-covered Pt at 0.5 μm field of view (FoV), under ideal SEM imaging conditions (very small primary beam spot size of 1 nm). (c) A linescan across the yellow line in (b), for 0.5 μm (black) and 50 μm FoV (blue), the latter representing typical view field used in this work. (d) A histogram depicting two distinct SE contrasts, clearly recognizable in the simulated image under ideal imaging conditions. (e-g) The results of simulations utilizing SEM parameters close to real experimental conditions (primary beam spot size of 40 nm). The increased spotsize used to generate SE image in (e) leads to much weaker image contrast, as evidenced by the linescan for both FoVs (f) and diminished second peak in the histogram (g). (h) Schematic representation of the SE simulation setup depicting 55 nm band composed of alternating 5 nm stripes of O and CO, approximating the composite inner structure of a reaction wave. Panels (i-k) and (l-n) show the resulting SE images, line profiles and reconstructed histograms for 1 nm and 40 nm spot size, respectively.

The simulation demonstrates the limits of work-function contrast imaging on dynamic oscillating systems under realistic beam settings. Although features as small as 5 nm are theoretically distinguishable (panels j and k), larger beam currents required to mitigate the signal-to noise ratio cause strong averaging of the SE signal, rendering images with indistinguishable nanoscale-size adsorbate phases.

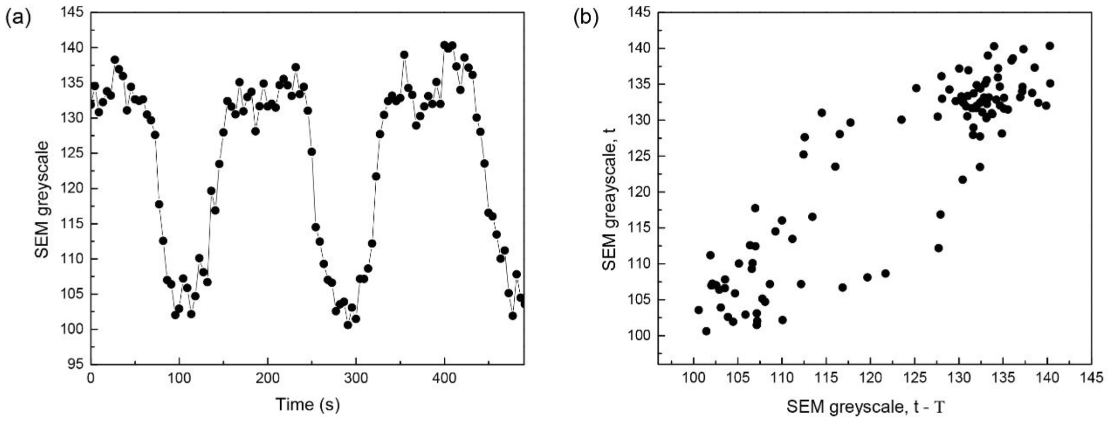

Fig. S2: SEM data analysis. (a) Time-trace reconstruction calculated from the wavefront propagation velocity of a greyscale signal derived from a spatial line scan of Tescan UHV-SEM SE images at p(CO) = 4×10^{-4} Pa, p(O_2) = 1.5×10^{-3} Pa. T = 204 °C. (b) Phase-portrait of data from (a) with time-delay τ corresponding to KPFM portraits from Fig. 3. In contrast to the KPFM-derived phase-portrait, SEM-derived one exhibits a nearly elliptical shape. This suggests nearly harmonic oscillations with similar forward and backward transitions, confirming the difficulty to distinguish fluctuations and gradients in wavefront adsorbate concentrations in real SEM imaging conditions.
